# Fine resolution mapping of population age-structures for health and development applications

**DOI:** 10.1098/rsif.2015.0073

**Published:** 2015-04-06

**Authors:** V. A. Alegana, P. M. Atkinson, C. Pezzulo, A. Sorichetta, D. Weiss, T. Bird, E. Erbach-Schoenberg, A. J. Tatem

**Affiliations:** 1 Centre for Geographical Health Research, Geography and Environment, University of Southampton, Highfield Southampton, UK; 2 Department of Zoology, University of Oxford, Oxford, UK; 3 Fogarty International Center, National Institutes of Health, Bethesda, MD, USA; 4 Flowminder Foundation, Stockholm, Sweden

**Keywords:** demography, geo-statistics, mapping

## Abstract

The age-group composition of populations varies considerably across the world, and obtaining accurate, spatially detailed estimates of numbers of children under 5 years is important in designing vaccination strategies, educational planning or maternal healthcare delivery. Traditionally, such estimates are derived from population censuses, but these can often be unreliable, outdated and of coarse resolution for resource-poor settings. Focusing on Nigeria, we use nationally representative household surveys and their cluster locations to predict the proportion of the under-five population in 1 × 1 km using a Bayesian hierarchical spatio-temporal model. Results showed that land cover, travel time to major settlements, night-time lights and vegetation index were good predictors and that accounting for fine-scale variation, rather than assuming a uniform proportion of under 5 year olds can result in significant differences in health metrics. The largest gaps in estimated bednet and vaccination coverage were in Kano, Katsina and Jigawa. Geolocated household surveys are a valuable resource for providing detailed, contemporary and regularly updated population age-structure data in the absence of recent census data. By combining these with covariate layers, age-structure maps of unprecedented detail can be produced to guide the targeting of interventions in resource-poor settings.

## 1. Background

Age is an important demographic variable that affects disease burden estimates [1] and mortality [2]. Defining the extent of public health need for specific age-groups and its distribution in space and time are critical to support interventions to combat disease burden, and plan and manage resources effectively. This includes interventions such as vaccination [3], insecticide-treated bednets (ITNs) for malaria as well as the delivery of healthcare to underserved populations [4]. Moreover, the production of health metrics [5,6] and spatial models of processes influenced by demographics [7,8] are increasingly reliant on spatial data on population age-structures. To support such efforts, quantitative information on the numbers or proportions of age-groups of interest in space and time is needed because these can vary significantly within and across countries.

Current methods of estimating population age-structures rely on census data. However, in most countries, population censuses are conducted every 10 years at best, and longer in many low-income countries. For example, the last population censuses conducted in the Democratic Republic of Congo, Somalia and Myanmar were in 1984, 1987 and 1983, respectively. Thus, census data can often be outdated, unreliable and provided at coarse spatial resolution [9], and estimates between censuses may not be accurate owing to changes such as migration that can be difficult to account for [10]. This makes it challenging for many government agencies and intervention programmes to use these data for efficient planning and delivery. Previous research that focused on quantifying progress towards development and health goals has often relied on simple national-level adjustments to obtain distribution maps of key denominator groups [11–13]. Detailed information on the distribution of age-structured population in space and time could therefore help optimize intervention planning, improve the measurement of key development and health indicators and produce spatial models that are reliant on demographics.

The past decade has seen marked growth in the regular implementation of national household surveys to provide important development and health measurements in the absence of reliable national reporting systems. There has also been an increase in the use of global positioning systems (GPS) in such surveys to enable the geo-referencing of information collected. These surveys, for example the demographic health surveys (DHS) [14], malaria indicator surveys (MIS) [15], living standard measurement surveys (LSMS) [16] and the multiple indicator cluster surveys [17], provide information on various demographic and health indicators between different low-income countries and across time. Moreover, the provision of GPS cluster centroid locations has enabled fine spatial resolution disease and poverty mapping using model-based geostatistical (MBG) approaches [18–21]. Such data therefore provide an opportunity to achieve more spatially detailed, accurate and regular estimates of age proportions to support the delivery of interventions, improve the precision of health and development metrics, and provide valuable base layers for spatial models.

Here, we demonstrate the fine resolution mapping of the under 5 years of age population proportions in Nigeria using three nationally representative surveys conducted between 2008 and 2010. The aim was not only to provide contemporary and spatially detailed 1 × 1 km grid cell estimates of the distribution of the population under the age of 5 years in 2010, but also to produce robust estimates of uncertainty around predictions. The outputs were compared with existing approaches for the production of age distribution spatial data. In addition, the differences obtained in using these existing approaches versus the household survey-derived estimates produced here for measuring the size of populations covered by ITNs and childhood vaccinations were explored.

## 2. Methods

### 2.1. Nigeria context

The study focused on Nigeria, the most populous country in Africa. It ranks as 153 of 182 countries on the human development index [22]. Like other countries in sub-Saharan Africa, Nigeria continues to experience high population growth at an average annual rate of 3.2% and is uncertain about achieving several of the millennium development goals (MDGs) [23]. Despite an improvement in gross domestic product [24], the majority of the population still live on less than US$1.25 per day and child mortality indicators are still short of the MDG targets with under-five mortality at 128 per 1000 live births (MDG target is 64 per 1000 live births) and infant mortality at 69 per 1000 live births (the MDG target is 30 per 1000 live births) [25]. Approximately 29% of children under the age of 5 years use ITNs [26]. The child health and nutrition indicators show that at least 25% of children were immunized (BCG, measles, DPT and Polio) in the first 2 years of life which indicates an increase of approximately 13% since the 2003 DHS [27]. Currently, the planning of vaccination strategies and needs, e.g. polio in the north of the country, is often based upon population counts projected forward using national growth rates from the 2006 census, and then an assumption of a uniform 20% of the population being under 5 years of age is used to adjust these totals to obtain subnational numbers.

### 2.2. Data

Data on the proportion of the population that is under 5 years of age were obtained from three nationally representative household surveys of Nigeria, namely the 2008 DHS [28], the 2010 MIS [26] and the 2010 LSMS-ISA panel [29]. These nation-wide cross-sectional surveys include modules enumerating the *de facto* members of the household. A household refers to a person or group of people related or unrelated that usually lives together in the same dwelling unit. The 2006 Nigeria household and population census provided the sampling frame for all the surveys. In each survey, a stratified two-stage sampling design was adopted where at the first stage clusters (census enumeration areas, EAs) were selected and stratified by urban and rural status. At the second stage, a random sample of households was selected from a household listing within the selected cluster [30]. Sampling was based on proportion-to-population size at the cluster-level such that the number of households varied in each state. Geographical locations of the selected cluster centroids in each survey were calculated. For all the surveys, a cluster centroid geolocation displacement was introduced at the processing stage to anonymize the cluster location. This was up to 5 km in rural areas and up to 2 km in urban areas, with a further 1% of rural clusters displaced up to 10 km [30]. Urban areas in Nigeria are officially defined based on settlements with populations of more than 20 000 [31]. The response data used in our analysis consist of cluster-level proportions of children less than 5 years old, calculated across all households in a cluster.

**Figure 1.**
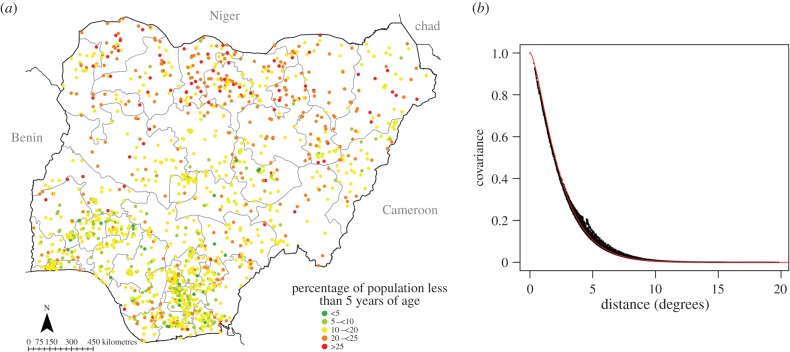
(*a*) The distribution of cluster-level data from the national representative household surveys (the DHS, MIS and LSMS-AIS) and (*b*) the associated covariance function from SPDE (black dots) for the data (*n* = 1624) with superimposed theoretical Matérn model (red line) showing only slight deviation beyond 550 km (or 5°). The *x*-axis shows the distance in degrees latitude and longitude, whereas the *y*-axis shows the covariance with scaling parameter log(*κ*) = −0.47(−1.07 − −0.46) (confidence interval) and smoothing parameter log(*τ*) = 2.85(2.42–2.85). The model calculated nominal range of influence on the *x*-axis was approximately 535 km. (Online version in colour.)

A spatial database combining the three surveys was established. Each record (*n* = 1624) was linked to administrative divisions, dates of survey and household population. Basic checks were applied to the merged dataset to investigate possible errors. For example, a consistency check was applied to the total population column in comparison with the respective age-structured columns. Geographical coordinates were checked by comparing the reported survey locations (administrative boundary) and actual map positions. Figure 1*a* shows the cluster locations coloured according to the proportions of under-fives, which exhibit spatial structure, as evidenced by the covariance function in figure 1*b*, which measures the spatial dependence.

### 2.3. Assembling plausible covariates for mapping the proportion of the population under 5 years

Predicting the under-five population proportions at locations without survey data requires exploiting both the spatial covariance structure in the survey data (figure 1*a*,*b*) and the relationships with covariates. Several socio-economic, physical (topographic, climatic and environmental) and political factors are associated with the varying distributions of demographics [21,32]. These factors affect (directly or indirectly) the distribution and growth of population. Favourable covariates that are available widely and measured consistently for modelling population are therefore land use or land cover, urbanization, vegetation indices, climatic conditions and socio-economic indicators [32,33]. However, these do not always correspond spatially or temporally to the respective dates of surveys. Thus, in this case, we assembled long-term means representing the climatic or environmental variables. Other covariates were derived from ancillary vector and raster datasets such as distance to roads, or major urban areas (a summary of assembled covariates is provided in the supplementary material).

### 2.4. Selecting a suitable set of covariates

The objective was to build a spatio-temporal model that uses a suitable combination of covariates to predict the proportion of population under 5 years at a fine spatial resolution. A two-stage process was used to arrive at a suitable model combination that best predicts the under-five population. First, covariates were selected via a non-spatial generalized linear regression model (*glm*) approach to identify suitable predictor variables (that are fewest in number and easily interpretable, with a predetermined relationship with the response variable [34]). Second, the selected set of covariates were then used in the Bayesian approach.

The use of many covariates may result in over-fitting especially where the data assembled are from observational studies based on different study designs, sampling considerations and sample sizes which are then combined to describe a random process [35]. Preliminary model selection of covariates that best describes the response is a widely accepted exercise in statistical modelling [34].

The choice of covariates should be guided by the principle of parsimony. There are several proposed approaches as reviewed by Murtaugh [34] including the widely criticized stepwise procedures (see [36,37] and references therein). Subset selection based on a statistical criterion, such as the Akaike information criterion, is the most commonly used in statistical modelling. Such criterion methods penalize model deviance (i.e. minus twice the log-likelihood) [38].

Covariate selection was implemented in the *bestglm* package in R using the leap algorithm [38]. Thus, a *glm* model with lowest Bayesian information criterion (BIC) was selected after covariates were regressed against the proportion of under-fives. In the BIC criterion, a uniform prior is usually imposed on all possible models.

### 2.5. Modelling the population proportion under five using model-based geostatistics

The application of geostatistics in environmental applications is well established, but little work has been undertaken in population distribution modelling. Early geostatistical applications were in geology and mining, although other applications can be found in a variety of disciplines [39,40]. These classical methods have developed rapidly since the 1960s in line with the emergence of statistical computer packages that can readily implement models. The geostatistical approaches exploit the spatial and temporal covariance in the data and relationships to covariates to generate posterior estimates while at the same time estimating uncertainty around these estimates [41].

The theory of regionalized variables, underlying geostatistics, allows each observation to be treated as being drawn from a distribution (usually Gaussian) that has a spatial extent, thereby extending the concept of a random variable *Z* to that of a random function (RF) *Z_u_* of space *u*. Thus, the RF *Z_u_* can have a series of outcomes (realizations) in space and relate to another point at a different location based on a function of distance (generally Euclidean distance) [42,43]. The RF has first-order stationarity if for any set *n* ≥ 1, the distribution of (*z*(*u*_1_), … ,*z*(*u_n_*)) is equal to that of (*z*(*u*_1_ + *h*), … ,*z*(*u_n_* + *h*)), where ***h*** is the lag vector in the two-dimensional spatial domain *D* ⊂ *R*^2^ [42,44,45]. For spatio-temporal models, the joint space–time formulation requires observations in space and time, based on RF *Z*(*s*, *t*)∈*D* × *T* (where *D* is the spatial domain and *T* is the temporal domain), separated by lag vector (*h*, *τ*), where *h* = *s* − *s*′ and *τ* = *t* − *t*′ refer to spatial and temporal lags, respectively [46].

Space–time geostatistical formulations with large datasets often result in the *big n problem* where estimating the covariance structure is of order *O*(*n*^3^) [47]. Here, the posterior approximations were produced using the integrated nested Laplace approximations (INLA) for latent Gaussian models [48,49]. INLA is faster computationally compared with Markov chain Monte Carlo algorithms that use sampling algorithms such as the Gibbs sampler or Metropolis–Hastings.

The outcome variable was the proportion of the under-five population, which was unevenly distributed in space and time. The methodology used data at known cluster centroid locations (geo-referenced using GPS), survey date, together with the selected set of covariates that aim to predict the proportion of the population that is under 5 years. The data and spatially matched covariates were then used in a Bayesian hierarchical spatio-temporal model, implemented through a stochastic partial differential equations (SPDE) approach with INLA for inference, to produce continuous maps of the estimated proportion of the population that is under 5 years old in each 1 × 1 km grid square in Nigeria. Table 1 shows the various model specifications based on different combinations of the selected covariates.

**Table 1.** Bayesian model specification based on covariates selected using non-spatial generalized regression.

	accessibility index (maximum)	EVI (mean)	land cover	night-time lights
model 1	x	x	x	
model 2	x	x	x	x
model 3		x	x	x
model 4	x		x	x
model 5	x	x		x

In the SPDE method, a Gaussian process model with Gaussian likelihood and link identity based on the linear predictor of proportion of the population that is under 5 years old was represented as a realization of a spatio-temporal process of the outcome variable at each cluster location, time of survey, covariates and measurement error defined by Gaussian white noise. The resulting space–time covariance matrix from the spatial and temporal domains informs the spatial range and temporal lag of the prediction model, so that observations have decreasing effects on the predictions with more separation in space and time.

In the SPDE approach, a continuous domain Gaussian random field (GF) was represented as a Gaussian Markov random field (GMRF). GMRFs result in sparse covariance matrices that are computationally faster. In this analysis, an SPDE with a stationary Matérn covariance was used. This model was applied to produce continuous predictions of the proportion of the population under the age of 5 years at 1 × 1 km spatial resolution for 2010 (full detail of model specification in the electronic supplementary material).

### 2.6. Model validation

Model selection was undertaken by comparing the deviance information criterion (DIC) and marginal likelihood of different models [50]. Validation was implemented in two steps. First, internal model validation was implemented by assessing calibration using a leave-one-out cross-validation approach [51]. The conditional predictive ordinate, which is the probability of observing a value given all other data, was examined for all observations [48]. Second, an external model validation procedure was applied based on a 10% subset of the data (*n* = 162). Predictions were made at validation locations and compared with the observations. The Pearson's product–moment correlation coefficient was computed to quantify the linear relations between observed and predicted values alongside the mean prediction error (MPE), mean absolute error (MAE) and root mean squared error (RMSE). The last two quantities assess bias and accuracy, respectively.

### 2.7. Application and comparison with existing approaches

The application focus was on two intervention needs, namely the distribution of ITNs for malaria prevention (see electronic supplementary material) and coverage of basic vaccination for childhood diseases. The posterior predictions of under 5 years of age proportions were multiplied with Nigeria population maps from the WorldPop project [21] to estimate the under-five population at 1 × 1 km spatial resolution and the 95% credible intervals for 2010. A separate similar analysis using the WorldPop estimate was repeated using the census estimates [31] (projected using the intercensual growth rate) and the UN under 5% estimate (medium scenario, 17.5%) [52] to extract two other under-five population maps that match with previous widely used derivations. Thus, the three under 5 years old population estimates (from MBG, census and the UN) were all derived from the same WorldPop estimate, meaning that differences in totals were solely attributable to the methods for estimating the under-five proportion, rather than overall population distribution or numbers. A similar approach was used to estimate intervention coverage on malaria prevention using ITNs based on the 2010 MIS and on the basic vaccination from the 2008 DHS. Basic vaccination, defined as one BCG vaccine against TB; three doses of DPT vaccine to prevent diphtheria, pertussis and tetanus (DPT); at least three doses of polio vaccine and one dose of measles vaccine, was assessed for children aged 12–23 months. Small area estimation approaches [53] were used in the analysis of the coverage of these interventions (population protected) at state (administrative 1) level (see the electronic supplementary material). Finally, the absolute and percentage differences in intervention coverage estimates between the census, the UN and the MBG-based approaches were summarized at state level to explore the scale of differences achievable through accounting for subnational population heterogeneities and the use of more contemporary data.

## 3. Results

### 3.1. Data summary

A summary of the assembled data from the three household surveys is provided in the electronic supplementary material. In total, 1624 unique clusters were assembled, and overall, the under-five population constituted the largest proportion of the survey (electronic supplementary material). The BIC approach yielded the following covariates: accessibility, night-time lights, land cover and enhanced vegetation index (EVI) as predictors of the proportion of those under the age of 5 years. A further exploratory analysis showed that some selected variables had a negative correlation (electronic supplementary material).

### 3.2. Model results

**Figure 2.**
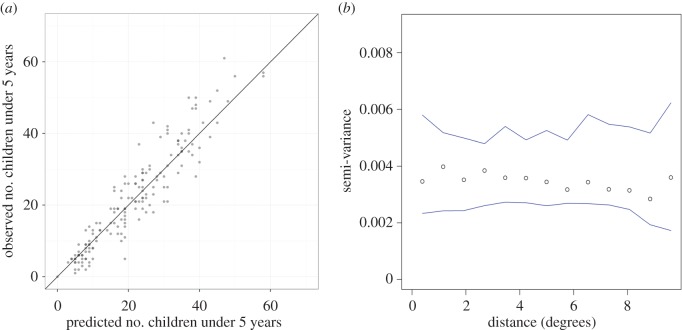
Validation plots showing. (*a*) Scatter plot of the association between the observed against predictions of the 10% subset data (*n* = 1624) and (*b*) semi-variogram plot (*y*-axis semi-variance and *x*-axis distance in degrees) and associated envelopes (minimum and maximum range expected by chance in the absence of spatial autocorrelation) of the standardized residuals. The semi-variogram is a measure of autocorrelation with distance. (Online version in colour.)

There was minimal difference between the three spatio-temporal models based on the DIC and the marginal likelihood (table 2). We elected to use model 2 (table 2) based on the DIC-marginal likelihood combination compared with the other four models. The prediction ability was assessed using the MAE as well as an assessment of prediction performance based on the 10% validation sample. The MPE for the model was very small (−0.00001), whereas the MAE was 0.03 and the RMSE was 0.04 (table 2). This indicated the average tendency to over-predict by 0.03. Pearson's correlation between observations and predictions was 0.63, and the corresponding scatterplot between the observations and predictions is shown in figure 2*a*. The analysis of residuals showed minimal autocorrelation as depicted in the semi-variogram of the residuals in figure 2*b*, indicating that most of the spatial structure was accounted for during the modelling exercise.

**Table 2.** Bayesian spatio-temporal model comparisons for the under-five population based on selected parameters and validation statistics. DIC, deviance information criteria; P_D_, number of effective parameter of the model; MPE, mean prediction error; RMSE, root mean square error.

	DIC	*P*_D_	marginal likelihood	MPE	MAE	RMSE	correlation
model 1	−4717.23	79.19	2271.73	−0.000013	0.0327	0.0427	0.6320
model 2	−4685.44	72.70	2254.245	−0.000014	0.0323	0.0424	0.6345
model 3	−4717.66	77.80	2272.611	−0.000017	0.0311	0.0408	0.6865
model 4	−4686.44	73.08	2261.950	−0.000012	0.0337	0.0436	0.6064
model 5	−4686.28	72.56	2262.600	−0.000013	0.0334	0.0434	0.6135

Table 3 shows the posterior distribution of the fitted model parameters including the fixed effects and random effects. The posterior mean of the intercept was 0.1815, showing that the overall predicted percentage of under-five population was approximately 18% before accounting for the various covariate effects. For accessibility, night-time lights and EVI, the marginal variance and the nugget were significant at the 95% credible interval, which confirmed the importance of these variables and parameters in prediction. The nugget effect was very small at 0.002 and the marginal variance from the Matérn covariance was also small (0.0007).

**Table 3.** Posterior distribution (mean, standard deviation and quantiles) of parameters for model 2.

parameter	mean	standard deviation	5%	50%	95%
intercept	0.1815	0.014	0.1593	0.1812	0.2047
accessibility index (maximum)	0.0044	0.0019	0.0013	0.0044	0.0076
EVI (mean)	−0.0045	0.0025	−0.0086	−0.0045	−0.0003
land cover	−0.0035	0.0024	−0.0076	−0.0035	0.0005
night-time lights	0.0016	0.0023	−0.0022	0.0016	0.0051
rho (time process) parameter (*ρ*)	−0.4699	0.3597	−1.072	−0.4636	0.1137
measurement error parameter	0.0022	0.0001	0.0021	0.0022	0.0024
the marginal variance	0.0007	0.0003	0.0003	0.0007	0.0014
model range (km)	534.6865	198.1813	280.5734	497.7561	911.8705

### 3.3. Predicted under-five proportions and comparison with existing estimates

**Figure 3.**
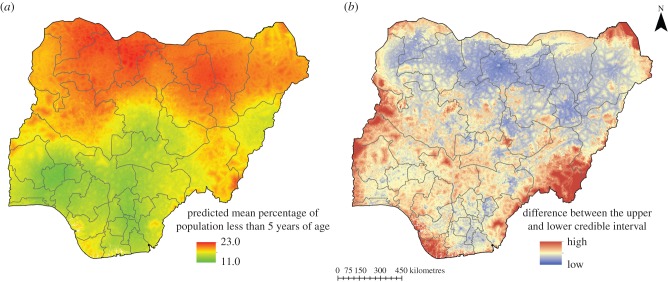
(*a*) Mean predicted percentage of population under the age of 5 years based on model-based geostatistics (*b*) map of differences (high and low) between the upper and lower limit of predictions (i.e. the 95% Bayesian credible intervals). (Online version in colour.)

**Figure 4.**
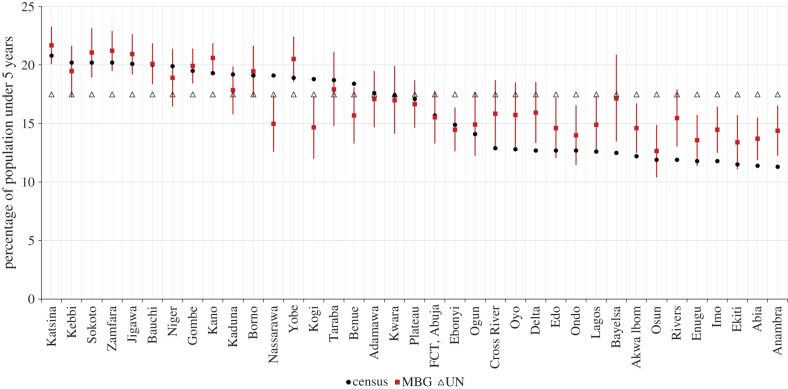
Plot of the estimated percentage of children under the age of 5 years in Nigeria (*y*-axis) by state (*x*-axis) from the three different estimates namely: the model-based geostatistics (MBG) approach (red rectangles with Bayesian prediction intervals), the projected census estimates (black circles) and a single UN estimate value for the whole of Nigeria (triangles). The plot has been ordered by the census estimates. (Online version in colour.)

Figure 3*a* shows the predicted proportions of the population under 5 years of age per 1 by 1 km grid cell from the geostatistical modelling, whereas figure 3*b* shows the difference between the upper and lower limits of prediction, highlighting the varying levels of uncertainty in the prediction outputs. In general, southern Nigeria showed lower proportions of children under the age of 5 years compared with the northern regions. For example, Kano, Katsina and Kaduna states had some of the highest proportions less than 5 years. Figure 4 shows a comparison of the percentage of the population under 5 years by state in Nigeria based on the three different estimates generated from adjustments of a total population gridded estimate using the MBG approach, UN national estimates and estimates derived from the 2006 census at state level. There were differences in subnational estimates generated depending on the approach used. The triangles in figure 4 are based on simple adjustment of population totals using the national-level UN estimate of 17.5% under the age of 5 years, i.e. not accounting for subnational variation (as undertaken in, for example, [11–13,54–57]). Significant subnational variation is, however, apparent when using either the 2006 census data (black circles) or the estimates produced by the MBG approach outlined here (in red), with some estimates below 12% and others over 20%.

### 3.4. Comparison of insecticide-treated bednets and vaccination coverage estimate variations

**Figure 5.**
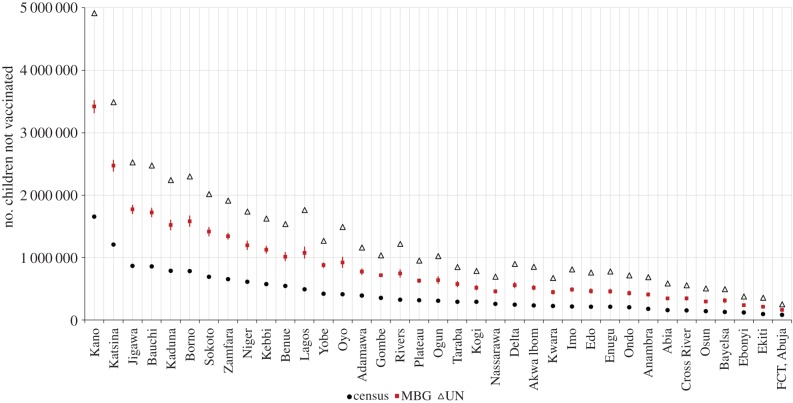
Comparison of the number of children not vaccinated (*y*-axis) by state (*x-*axis) from the three different estimates namely: the model-based geostatistics (MBG) approach (red rectangles with Bayesian prediction intervals), the projected census estimates (black circles) and UN estimates for the whole of Nigeria (triangles). (Online version in colour.)

**Figure 6.**
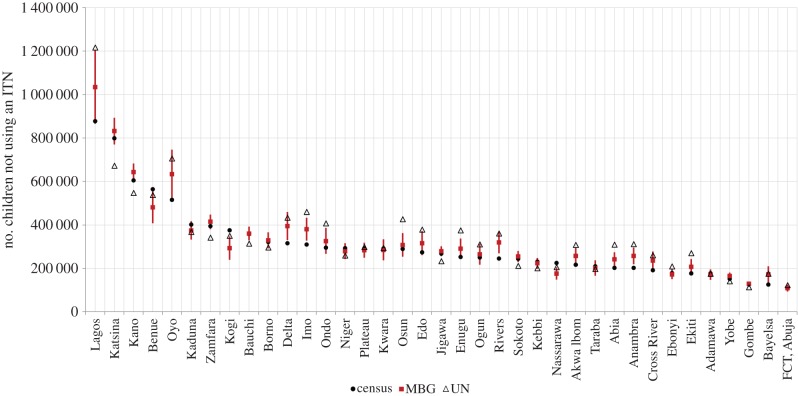
Comparison of the number of children not using an ITN (*y*-axis) by state (*x-*axis) from the three different estimates namely: the model-based geostatistics (MBG) approach (red rectangles with Bayesian prediction intervals), the projected census estimates (black circles) and UN estimates for the whole of Nigeria (triangles). (Online version in colour.)

The effects of the above variation can be seen in the production of intervention coverage estimates at a national level. The 2008 DHS showed that about 39.7% of children between 12 and 23 months received basic vaccination in Nigeria (i.e. 60.3% of children not vaccinated) with higher rates of coverage in the south. Maps of vaccination coverage are included in §4 of the electronic supplementary material. Figure 5 shows a comparison of the number of children not vaccinated by state based on the MBG estimates developed here, in comparison with the UN or the census-derived under-five population datasets. The widest gap in vaccination was in Kano, Katsina and Jigawa. Similar results were obtained for children not using ITNs (figure 6). This variation suggests that accounting for fine-spatial resolution subnational variation can produce sizeable differences in estimated metrics.

## 4. Discussion

This study used data from three household surveys in Nigeria to quantify the proportion of population under the age of 5 years using a Bayesian hierarchical spatio-temporal model. The survey data show that considerable subnational variation in the population age-structure exists. Much recent and influential research on global disease burden [12], estimating MDG indicators [11,57], quantifying populations at risk [13,54,55] or mapping interventions [58], has been limited to simply using a national-level adjustment of population estimates to represent age-structures in the absence of more accurate, spatially detailed and reliable data. Results from this study suggest that detailed and contemporary depictions of population age-structures can be produced from survey data and mapped at fine spatial resolution. The fine spatial resolution estimates are simpler to integrate with gridded population total estimates that are commonly produced at the same spatial resolutions [9,21] and can be summarized readily to policy-relevant administrative units for planning, decision-making and resource allocation. Second, these contemporary estimates of population age-structures can be produced without reliance on census data that can be outdated and unreliable in many countries, and with quantification of uncertainty. Moreover, the use of covariates not only enhances the scientific understanding of associations with potential driving factors of population age-structures, but can also be applied in other countries because of their wide and consistent coverage. In this study, land cover, night-time lights, accessibility and a vegetation index emerged as important covariates over more societal-based indicators (electronic supplementary material), with each either directly acting on, or acting as a surrogate for, factors influencing population age-structures. Moreover, the quantification of uncertainty here has additional advantages in guiding the positioning of future surveys to optimize mapping accuracy and hence enhance understanding of age-structures.

Age is a central variable in the fields of development, humanitarian response, epidemiology and public health. Certain age groups are more vulnerable to economic fluctuations [59], conflicts [60] and natural disasters [61], whereas health events vary with age [62]. The international development agenda in the past two decades has been shaped by two themes. The first on achieving the eight MDGs by 2015 [63]. The second on achieving the upcoming sustainable development goals (SDGs) focusing on sustainable cities and human settlements, climate change, societal protection and biodiversity among other numerous goals [64]. Mapping has increasingly been used in estimating indicators [5,65], assessing progress on some of these goals [58,66], as a basis for spatial modelling [67–69], and shaping policy on health and development [33,68]. However, despite major advances in the mapping of the prevalence of development metrics and health outcomes, many applications in the most resource-poor settings still rely on national-level estimates of age proportions from the UN (or other producers of demographic statistics), or outdated census data of coarse spatial resolution, to provide denominators for conversion of prevalence estimates to numbers at risk [13,54]. To support health and development modelling efforts, government assessments of need, and measuring progress towards meeting the MDGs and SDG targets requires reliable and contemporary spatial baseline data on the population and its age-structure to construct relevant policies as well as estimate outcomes accurately. GPS-located national household survey data provide a valuable new source of subnational demographic information that is more readily and regularly available than census-based estimates, and has the potential to be integrated with census data, where complimentary data features exist. Here, we have shown how such geolocated survey cluster data can be used to build contemporary and detailed datasets on population age-structures with full quantification of model-based estimates of uncertainty.

Many government programmes, multilateral and bilateral agencies require disaggregated estimates with associated confidence intervals for budgeting and planning purposes [70]. An important finding here suggests that the current practice in many applications of using national-level age proportion metrics likely under-predicts the proportion of the population under the age of 5 years substantially in the most poor and highly burdened populations. For example, there were substantial differences in the intervention coverage metrics when estimated using the fine spatial resolution model-based approach compared with use of census or national-level estimates. While coverage of interventions can differ substantially between urban and rural populations [71], the large differences in under-five age-structure estimates can result in under-estimation or over-estimation of needs. This also applies to other sectors, such as development or economic indicators and disaster relief where these metrics are required and used widely. While we have focused here on estimation of the denominator, measurement of the numerator is equally important in arriving at accurate coverage estimates. In some settings, the quality of the data on the numerator or scarcity of it makes the numerator the factor contributing the greatest uncertainty to coverage estimates, whereas in other settings, the opposite is true.

Some limitations remain, however. First, we had no control over data coverage and content errors given that these were managed from different systems. Such errors relate to misclassification in household data or covariates such as land cover, and recording and data entry errors. While the model performance was satisfactory, some sources of errors contributed to model uncertainty, and unexplained variance remains. For instance, inherent in the DHS and LSMS data are the displacements of cluster locations for protection of respondent population anonymity [72], and this may result in two types of errors. The first may result in incorrectly linking the covariate to age-structure owing to mismatch between the scale of displacement and covariate spatial resolution. We mitigated this error source by defining buffers around the survey locations during covariate extraction, which also theoretically improved the spatial representation of a cluster. In urban areas, in addition to the displacement issue, covariates available at a national level do not measure within-urban variability well. For the second problem, this meant that urban areas were generally predicted with the same homogeneous values, rather than being able to discern within-city variation. Upcoming data products, such as the human settlement layer from the Joint Research Centre (JRC) [73], may mean that within-city variation can be better represented in the future, and ongoing work is exploring the effects of cluster displacement and refined covariate layers. We mainly used cross-sectional rather than used longitudinal data here, with the latter being more advantageous for tracking change over time. Although the modelling set-up accounted for different survey dates, this was not sufficient to be able to interpret the nature of time-series patterns impacting on population as indicated by the AR(1) *ρ* coefficient. The LSMS-ISA repeated survey of 2013 was longitudinal in design. However, a critical evaluation between the data used here and the follow-up survey did not show a significant change in demographic pattern to alter the distribution predicted here. Moreover, the sample sizes used in the LSMS-ISA were smaller when compared with the DHS. There still exists a lack of approaches for handling cluster weights in the type of model-based approach used here. However, first, there was minimum difference in the DIC (−4695.50) or marginal likelihood (2248.18) when cluster weights were incorporated as random effects compared with current results (table 2). Second, with the approach outlined here, the Gaussian white noise specified in the SPDE approach adds extra parametrization to the realizations of the unobserved levels of the proportions of the population. The space–time covariance matrix informed the spatial range and temporal lag of the predictions. Outside of the spatial and temporal range, the autocorrelation of the data becomes almost null. Lastly, there is a potential error introduced as a result of mismatch in the date of the survey and covariates. Long-term annual means were used for covariates, because most are not usually produced on a monthly basis or even annually.

The work presented here demonstrates the value of the combination of geolocated household survey data with spatial covariates in a Bayesian geostatistical framework for improving the quantification of the under 5 years of age proportion distributions in resource-poor settings where alternative reliable and contemporary data are unavailable, and points the way to a range of future innovations. First, the extension of this work through multinomial methods should enable the prediction and mapping of full population age-structures. Moreover, the linkage with increasingly sophisticated approaches for the fine-resolution mapping of population counts [74,75], will enable more accurate and contemporary estimates of total numbers at risk, particularly using approaches based on ‘bottom-up’ methods that use settlement extraction from fine spatial resolution satellite sensor imagery to estimate population sizes directly in the absence of census data. With geolocated household surveys measuring age-structures now being undertaken regularly, particularly in the most resource-poor countries, the potential also exists to undertake regular updates and monitor change at a global scale—something that has not previously been possible using decadal census data. Finally, the potential exists for the construction of hybrid approaches that can integrate the more regularly undertaken national household survey data with population census data, where reliable and recent data exist, and even novel data sources, such as mobile phone call data records, which have shown potential in demographic mapping [76].

A rising international focus on inequalities and the mapping of health and development indicators in the poorest parts of the world requires a strong evidence base with explicit quantification of uncertainties to ensure that data deficiencies are communicated effectively. In many low-income countries, we still have a poor understanding of the numbers, distributions and demographics of populations [9] and geolocated national household surveys are helping to improve this situation. The approaches outlined here make use of these data to provide robust estimates in unsampled locations and provide valuable data on key population groups, capturing the substantial demographic variabilities that can translate into improved health and development metrics.

## Supplementary Material

Response to Reviewers.doc

## Supplementary Material

Supplimentary Information (SI)
