# Supplementary material for: Fine resolution mapping of population age-structures for health and development applications
Source: J R Soc Interface. 2015 Apr 6;12(105):20150073. doi: 10.1098/rsif.2015.0073 (PMC4387535; doi:10.1098/rsif.2015.0073)
Supplement: Supplimentary Information (SI) [file rsif20150073supp2.doc]

**Fine resolution mapping of population age-structures for health and development applications in low income countries**

Alegana VA, Atkinson PM, Pezzulo C, Sorichetta A, Weiss D, Bird T, Erbach-Schoenberg E and Tatem AJ

**Supplementary information (SI)**

Table of Contents

[1. Data summary 2](#__RefHeading___Toc410469244)

[2. Covariates and Bayesian model specification 2](#__RefHeading___Toc410469245)

[2.1 Covariates 2](#__RefHeading___Toc410469246)

[2.2 Bayesian model specification for modelling the proportion of the population under 5 years of age 5](#__RefHeading___Toc410469247)

[3. Results of covariate selection procedure 6](#__RefHeading___Toc410469248)

[4. Estimating ITN use in children under five years in 2010 and vaccination coverage for children 12-23 months in 2008 in Nigeria 7](#__RefHeading___Toc410469249)

# Data summary

Table 1.1 provides a summary of the assembled data from the three household surveys namely: the Demographic Health Surveys (DHS 2008), the Malaria Indicator Surveys (MIS 2010) and the Living Standard Measurement Surveys (LSMS 2010). On average the population structure depicts a young population with 45% of population below the age of 15 years.

Table 1.1: Description of the data assembled in Nigeria by survey type, residence and administrative level 1

|  | **Number of Clusters** | **Number of Households** | **Mean Household size** | **Sample Population** | **Sample Under-fives** |
| --- | --- | --- | --- | --- | --- |
| **Survey** |  |  |  |  |  |
| DHS | 886 | 33,986 | 38 | 154,946 | 26,824 |
| LSMS | 499 | 4,993 | 10 | 27,426 | 4,116 |
| MIS | 239 | 5,890 | 25 | 30,134 | 6,074 |
| **Residence** |  |  |  |  |  |
| Urban | 522 | 14,261 | 27 | 62,697 | 10,110 |
| Rural | 1,102 | 30,608 | 28 | 149,809 | 26,904 |
| **State** |  |  |  |  |  |
| Abia | 41 | 1,080 | 26 | 4,354 | 614 |
| Adamawa | 42 | 1,196 | 28 | 6,575 | 1,154 |
| Akwa Ibom | 47 | 1,265 | 27 | 5,668 | 800 |
| Anambra | 54 | 1,261 | 23 | 5,018 | 752 |
| Bauchi | 50 | 1,304 | 26 | 7,759 | 1,671 |
| Bayelsa | 33 | 1,071 | 32 | 4,152 | 740 |
| Benue | 48 | 1,250 | 26 | 6,562 | 1,166 |
| Borno | 54 | 1,379 | 26 | 7,272 | 1,454 |
| Cross River | 41 | 1,072 | 26 | 4,816 | 804 |
| Delta | 46 | 1,265 | 28 | 4,911 | 770 |
| Ebonyi | 43 | 1,186 | 28 | 5,555 | 907 |
| Edo | 39 | 1,129 | 29 | 5,176 | 820 |
| Ekiti | 35 | 1,112 | 32 | 4,080 | 574 |
| Enugu | 46 | 1,173 | 26 | 4,896 | 637 |
| FCT, Abuja | 30 | 967 | 32 | 4,101 | 641 |
| Gombe | 36 | 1,098 | 31 | 6,392 | 1,339 |
| Imo | 53 | 1,210 | 23 | 4,812 | 622 |
| Jigawa | 41 | 1,159 | 28 | 6,449 | 1,325 |
| Kaduna | 44 | 1,274 | 29 | 7,479 | 1,341 |
| Kano | 61 | 1,630 | 27 | 9,259 | 1,906 |
| Katsina | 49 | 1,339 | 27 | 7,803 | 1,783 |
| Kebbi | 36 | 1,076 | 30 | 5,772 | 1,060 |
| Kogi | 43 | 1,264 | 29 | 5,313 | 773 |
| Kwara | 39 | 1,075 | 28 | 4,613 | 739 |
| Lagos | 62 | 1,748 | 28 | 6,836 | 980 |
| Nasarawa | 34 | 1,031 | 30 | 5,649 | 881 |
| Niger | 49 | 1,261 | 26 | 7,158 | 1,372 |
| Ogun | 39 | 1,143 | 29 | 3,782 | 595 |
| Ondo | 42 | 1,211 | 29 | 4,787 | 673 |
| Osun | 47 | 1,275 | 27 | 4,934 | 645 |
| Oyo | 55 | 1,402 | 25 | 5,143 | 787 |
| Plateau | 41 | 1,187 | 29 | 6,171 | 1,010 |
| Rivers | 54 | 1,375 | 25 | 5,214 | 804 |
| Sokoto | 37 | 1,151 | 31 | 5,877 | 1,287 |
| Taraba | 37 | 1,106 | 30 | 6,374 | 1,128 |
| Yobe | 41 | 1,130 | 28 | 6,281 | 1,323 |
| Zamfara | 35 | 1,014 | 29 | 5,513 | 1,137 |
|  |  |  |  |  |  |
| **Total** | **1,624** | **44,869** | **28** | **212,506** | **37,014** |

# Covariates and Bayesian model specification

## 2.1 Covariates

This section provides an overview of the assembled covariates used in the analysis. These are also summarised in Table 2.2 (page 4) which also shows the source. Four covariates were selected for being used in the geostatistical model, namely accessibility to major cities, location of night-time lights, quantification of vegetation and land cover.

**Accessibility index:** An index describing ‘accessibility’ per 1x1km grid cell, measured as estimated travel time to the nearest major settlement, was obtained for Nigeria [1]. Major settlements were defined based on having a population size of more than 50,000.

**Remotely-sensed satellite based covariates (EVI, Land cover and Nighttime lights):** For environmental covariates, enhanced vegetation index (EVI) a measure of photosynthetic activity ranging from 0 (no vegetation) to 1 (complete vegetation) was derived from Moderate-resolution Imaging Spectroradiometer (MODIS) sensor imagery [2-4] using an approach described in Weiss et al. (2014) and then summarized to produce a synoptic annual product. Levels of urbanicity were also derived from MODIS. There have been attempts linking the nighttime lights to population and human activity [5]. Here, the nighttime lights were derived from Visible Infrared Imaging Radiometer Suite (VIIRS)[6]. Land cover data were obtained from MERIS GlobCover [7], which classifies all 300m resolution grid cells into 22 classes similar to those used by the United Nation’s Land Cover Classification System (UN-LCCS) [8]. This was also resampled to 1x1km resolution.

**Other covariates:** Other covariates initially considered in the analysis include (i) a “distance to roads” map, generated using Open Street Map (OSM) road data [9]; (ii) a map of Nigerian settlement locations derived from Landsat Enhanced Thematic Mapper (ETM) images [10, 11]; (iii) the MODIS 500-m global map of urban extent [12, 13]; (iv) a map of conflict locations extracted from the Armed Conflict Location and Event Dataset (ACLED) [14]; and (v) the map of Geo-Referenced Ethnic Groups (GREG) [15]. .

Data preparation included not only resampling of raster grids to a common spatial resolution, but also projecting to a common geographic system and deriving buffers around the cluster locations to be used as extraction extent. Vector datasets were also rasterised to same resolution as other grid surfaces. The buffers were generally designed to account for the displacement introduced in the DHS and LSMS data (i.e. up to 2 km for urban clusters and up to 5 km for rural based clusters although and additional 1% of the rural clusters may be displaced by up to 10km) but not necessarily covering the physical areal extent of a village or cluster. The extraction of covariates was done at each cluster location (*n=*1,624) using the ArcGIS 10.1 Spatial Analyst extension [17] and the Geospatial Modelling Environment [18, 19]. Minimum, maximum, mean and standard deviation values were extracted from the continuous raster datasets, while modal values were extracted from the categorical ones.

Table 2.2 Summary of the assembled covariates

| **Covariate description (Unit)** | **Year** | **Category** | **Format/Type** | | **Spatial resolution (Degree)** | | **Source (URL)** |
| --- | --- | --- | --- | --- | --- | --- | --- |
| Distance to populated places with more than 50k people (hour) | 2000 | Accessibility | Raster/ continous | 0.0083 | | European Commission Joint Research Centre (http://bioval.jrc.ec.europa.eu/products/gam/download.htm) | |
| VIIRS night-time lights (nano-Watts/(sqcm*sr)) | 2012 | Night-time lights | Raster/ continous | 0.0042 | | NOAA (http://ngdc.noaa.gov/eog/viirs.html) | |
| MODIS Mean Enhanced Vegetation Index (N/A) | 2000-2012 | Vegetation | Raster/ continous | 0.0083 | | "Malaria Atlas Project" (MasterGrids Archive) | |
| Distance to settlements (meter) | 2010 | Distance | Raster/ continous | 0.0083 | | Input data from "WorldPop Project" (http://www.worldpop.org.uk) | |
| Distance to roads (meter) | 2014 | Distance | Raster/ continous | 0.0083 | | Input data from OSM (http://extract.bbbike.org/) | |
| MODIS-based Global Urban Extent (N/A) | 2000-2001 | Urban/Rural | Raster/ categorical | 0.0042 | | SAGE (http://sage.wisc.edu/people/schneider/research/data.html) | |
| Conflict locations (N/A) | 2010-2013 | Conflicts | Vector/ Categorical | - | | ACLED (http://www.acleddata.com/data/version-4-data-1997-2013/) | |
| Geo-referenced ethnic groups (N/A) | 2010 | Ethnicity | Vector/ Categorical | - | | ETH (http://www.icr.ethz.ch/data/other/greg) | |
| MERIS global land cover classification (N/A) | 2009 | Land cover | Raster/ categorical | 0.0028 | | ESA "GlobCover Project" (http://due.esrin.esa.int/globcover/) | |

## 2.2 Bayesian model specification for modelling the proportion of the population under 5 years of age

The interest here was to predict the response at locations not sampled and incorporating uncertainty into predictions. A Bayesian hierarchical spatio-temporal model was implemented in R-INLA [9-11] to produce a continuous map of proportion of the population under 5 years of age at 1 x 1 km spatial resolution using the cluster locations (longitude and latitude), date of survey and set of covariates. The Gaussian Function (GF) in INLA is represented as a Gaussian Markov Random Function (GMRF). Computations in INLA are carried out using the GMRF by approximating a set of spatial-temporal random function with weighted sum of basis functions. The advantage of computation using the GMRF as approximations to GF with Matérn covariance is due to the Markovian property of the former resulting in sparse matrices that are computationally efficient. The explicit link between the continuous domain GF is a solution to the stochastic partial differential equation (SPDE) expressed as:

$$\frac{\partial}{\partial t}\left(k^{2}-\Delta\right)^{\alpha/2}\left(\tau x(s,t)\right)=w(s,t)$$

Where $\left(k^{2}-\Delta\right)^{\alpha/2}$ is a differential operator, k is a scaling parameter, $\Delta$ is the Laplacian, $\alpha$controls the smoothness of realization, $\tau$controls the variance and $x(s,t)$is the spatial-temporal domain for s(s_1_,……,s_n_) locations. $w(s,t)$is the Gaussian white noise. A stationary model was implemented. The link between Matérn smoothness $\nu$and variance $\sigma^{2}$is given by $\alpha=\nu+d/2$where *d* is spatial dimension and marginal variance$\sigma^{2}=1/\left(\Gamma(\nu)\Gamma(\alpha)(4\pi)^{d/2}k^{2\nu}\tau^{2}\right)$. The above SPDE is solved via finite element method by triangulating the domain of interest. The SPDE is projected to a basis representation,

$$x(s,t)=\sum_{k} \psi_{k}(s,t)x_{k}$$

Where $\psi_{k}(s,t)=\psi_{i}^{s}(s)\cdot\psi_{j}^{t}(t)$a product of spatial and temporal basis function

Several advantages arise from such construction. First, the aforementioned resulting sparse covariance matrix is computationally efficient since GMRF is only discretely indexed at locations *x*_1_,……*x*_k_. Secondly the covariance function is flexible within the general family of covariances. The stationary Matérn covariance function for spatial lag distance$h=\left\|s_{1}-s_{2}\right\|$, s_1_ and s_2_ locations is expressed as:

$$C(s_{1}s_{2})=\frac{\sigma^{2}}{2^{v-1}\Gamma(\nu)}\left(kh\right)^{\nu}k_{\nu}\left(kh\right)$$

Where $k_{\nu}$ is the modified Bessel function of second order and $h$ is the Euclidean distance while $\sigma^{2}$is the marginal variance.

The SPDE generates precision matrix of form $Q=Q_{t}\otimes Q_{s}$where $Q_{s}$is the precision in the spatial domain and $Q_{t}$in temporal domain with $Q_{s}=\tau^{2}\left(k^{4}C+2kG_{1}+G_{2}\right)$ where C, G_1_ and G_2_ are sparse matrices while $Q_{t}$precision was based on a temporal AR(1) process similar to Camelletti et al. (2012).

A linear model was implemented using a Gaussian likelihood for the proportion of under-five population.

$$z(s,t)=x(s,t)\beta+\zeta(s,t)+\varepsilon(s,t)$$

Where $z(s,t)$are realizations of the process linked to a spatio-temporal structured predictor in an additive way, $x(s,t)$denotes set of covariates with $\beta$coefficients and $\varepsilon(s,t)$is the measurement error while $\zeta(s,t)$represent first order autoregressive dynamics with spatially correlated innovations. The AR(1) model for a Gaussian vector $x_{i}$is defines as $x_{i}=\rho x_{i-1}+\varepsilon_{i}$where the coefficient and $\varepsilon\sim N(0,\tau^{-1})$, $i=2,...,n$. The coefficient controls the time series pattern in ARIMA models [12]. The posterior distribution given observations and the parameters $\theta$is:

$$\left(y|x,\theta\right)\sim p(y|\eta_{,}\theta)$$

where $\eta$is the linear predictor given as linear combination of covariate, observations$y$and $\theta$parameters.

$$\eta=fixed(\beta)+f(time)+f(random)$$

The full model specification was given as

$$Z|\eta\sim GP\left(\theta,\sigma^{2}\psi(d_{i}\Sigma)\right)$$

Intercept $\pi(\beta_{0})\alpha1$

Fixed effects $\beta_{1}\sim N(0,1e-05)$

$w\sim N(0,Q(k,\tau))$, $\log\tau\sim N(m_{\tau},l_{\tau}^{2})$,$\log k\sim N(m_{k},l_{k}^{2})$

Where $m_{k}$range is 1/5 of domain and $m_{\tau}$was selected to have a standard deviation of 1.

# Results of covariate selection procedure

The assembled set of covariates (Table 2.1) was selected through the BIC approach described in the main text. The coefficients and 95% confidence intervals of the best-fit covariates from the total-set analysis are shown in Table 3.1.

Table 3.3 Results of the covariate selection.

Covariate selection analysis for Nigeria under-five population modelling showing the regression coefficients and the *p*-values of the best-fit covariates

| **Covariates** | **BIC** | | |  | **BIC*g*^1^** | | |
| --- | --- | --- | --- | --- | --- | --- | --- |
|  | **Coefficient** | **Standard error** | ***p*-value** |  | **Coefficient** | **Standard error** | ***p*-value** |
| Accessibility index (Maximum) | 0.0052 | 0.0014 | <0.001 |  | 0.0054 | 0.0014 | <0.001 |
| Nighttime lights (mean) | 0.0038 | 0.0013 | <0.001 |  | - | - | - |
| Enhanced Vegetation Index (EVI) (mean) | -0.0178 | 0.0014 | <0.001 |  | -0.0179 | 0.0014 | <0.001 |
| Conflict | - | - | - |  | - | - | - |
| Distance to population centre | - | - | - |  | - | - | - |
| Distance to road | - | - | - |  | - | - | - |
| MODIS urban areas | - | - | - |  | - | - | - |
| Ethnicity | - | - | - |  | - | - | - |
| Land Cover/Land Use | -0.0081 | 0.0015 | <0.001 |  | -0.0084 | 0.0015 | <0.001 |

1. BIC*g* used uniform prior on possible models of a fixed size (see Mcleod 2008)
2. Blanks indicate covariates not selected

The analysis of correlation of the selected variable is shown in Table 3.4 with most variables showing a negative correlation.

**Table 3.4 Pearson c**orrelation of the selected variables

|  | **Accessibility (towns) maximum** | **Mean EVI** | **Land cover (Globcover)** | **Night-time lights (viirs)** |
| --- | --- | --- | --- | --- |
| **Accessibility (towns) maximum** | 1 |  |  |  |
| **Mean EVI** | 0.26 | 1 |  |  |
| **Land cover (Globcover)** | -0.27 | -0.30 | 1 |  |
| **Night-time lights (viirs)** | -0.04 | 0.02 | 0.02 | 1 |

# Estimating ITN use in children under five years in 2010 and vaccination coverage for children 12-23 months in 2008 in Nigeria

The MIS data for 2010 were used in modelling the ITN use in children while the DHS data for 2008 were used to model the children vaccination coverage. The individual-level information was summarised to cluster level. Therefore, each cluster had aggregated information on total number of children under the age of five years who used an ITN the night before the survey (from MIS), and the summary information on number of doses received for BCG, DPT, polio and measles by children 12-23 months (from DHS).

A Bayesian linear mixed model approach was used to estimate ITN use at state level. The model had the form

$$Y_{i} = X_{i}\beta+\delta_{s}+ \varepsilon_{i} , \varepsilon_{i} \sim N (0,\sigma^{2}) ,$$

for observations $Y_{i} , i = 1 , .... , n ,$ and a vector of $X$ covariates. However, no covariates were used in both ITN and vaccination analysis to avoid overfitting. Spatial information was incorporated using a conditional autoregressive parameter $\delta_{s}$ [13]. A neighbourhood matrix was built from the state spatial units such that 1 was used for a unit $j,$ as neighbour or 0 otherwise. Finally a random slope parameter was included at the state level with zero mean and large variance. Figure 4.1 shows the population weighted averages of ITN use and vaccination coverage.


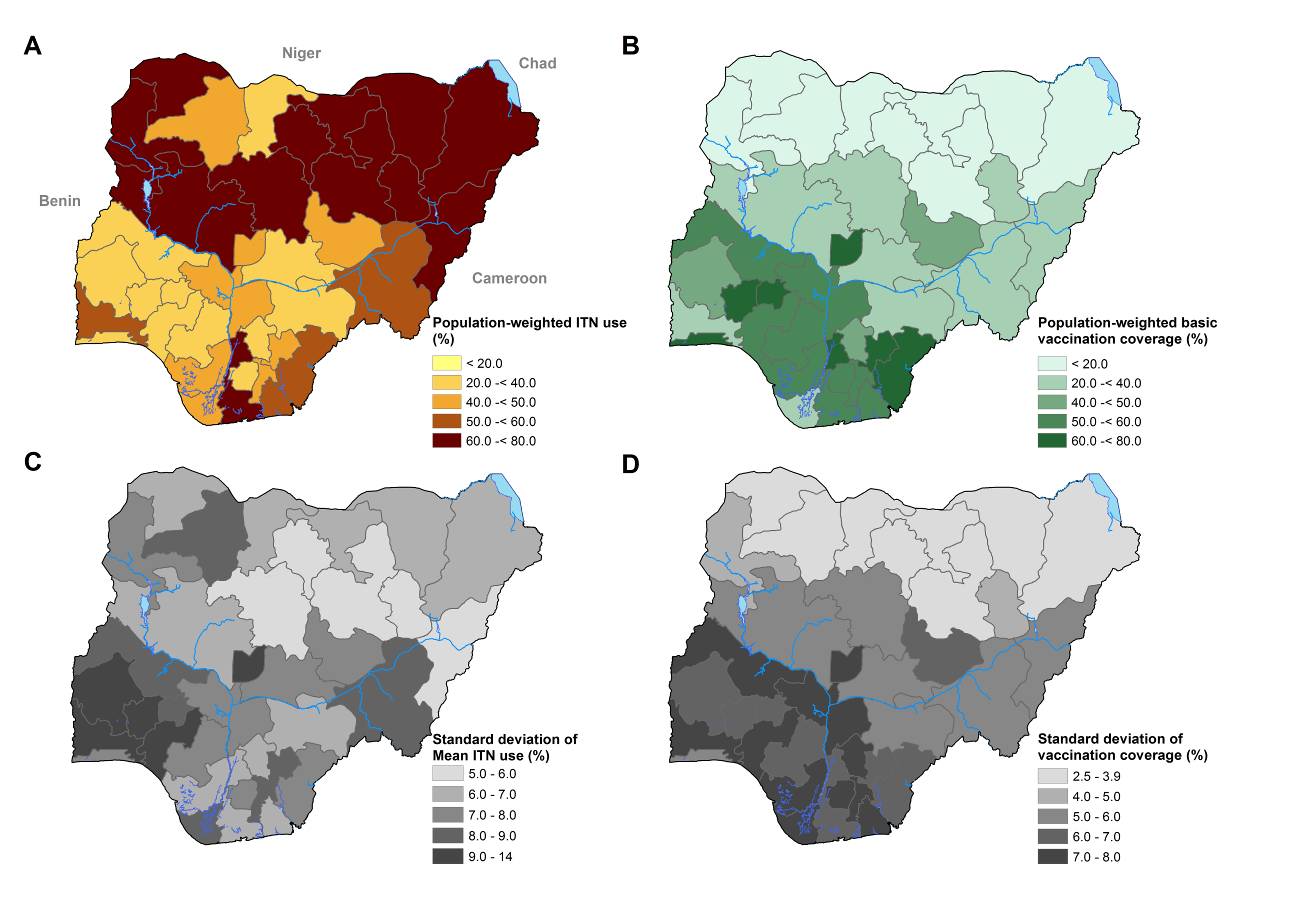


Figure 4.1 Estimates of ITN use and vaccination coverage.

Population-weighted means generated based on the estimated proportion of population under five years covered by interventions for **A)** Population-weighted mean ITN use in based on the 2010 MIS, **B)** Population-weighted mean basic vaccination coverage based on the 2008 DHS. Basic vaccination defined as one BCG vaccine against TB; three doses of DPT vaccine to prevent diphtheria, pertussis, and tetanus (DPT); at least three doses of polio vaccine; and one dose of measles. Standard deviation maps for ITN use and vaccination coverage shown in **C** and **D** respectively.

Table 4.1 to 4.3 provides summaries at national level (Table 4.5) and by state (Table 4.6, Table 4.7 and Table 4.8) on mean estimated ITN use and basic vaccination coverage based on the three derived population surfaces using the model-based geostatistical approaches (MBG), census and UN respectively. Estimate at national level varied (Table 4.5). At state level wider differences were shown based on the UN mean national estimate of proportion of population under five years old. The result suggested that using a single national estimate could result in unrealistic estimates at state level. For example, in Kano state, based on the MBG approach, approximately 644,265 children were not protected by ITNs in 2010 compared to 604,983 and 548,411 children from the census and UN derived estimates respectively. In terms of vaccination, in Kano, MBG estimated 1,763,286 children 12-23 months were not vaccinated compared to 1,655,776 from census and 1 500,943 from UN derived estimate.

Table 4.5: Estimates for under-five population at risk of malaria in millions in different endemicity classes and intervention coverage based on extract from MBG, the UN and the national census.

|  |  | | **PAR malaria^1^** | | | |  |  |
| --- | --- | --- | --- | --- | --- | --- | --- | --- |
|  | **Total Under 5 Population (millions)** | **<5%** | | **5%-10%** | **10%-50%** | **>50%** | **Estimated Number (millions) of Under 5 using ITN** | **Estimated Number (millions) of Children 12-23 months who received basic vaccination^2^** |
| Census | 24.21 | 0.05 | | 0.11 | 20.40 | 3.64 | 12.33 | 9.61 |
| United Nations | 26.43 | 0.05 | | 0.11 | 22.48 | 3.78 | 13.46 | 10.49 |
| MBG | 25.64 | 0.05 | | 0.11 | 21.71 | 3.77 | 13.05 | 10.18 |

1. Population under-five at risk of malaria based on age corrected parasite prevalence data for children between 2 to 10 years in Nigeria in 2010 [14].
2. Basic vaccination defined as one BCG vaccine against TB; three doses of DPT vaccine to prevent diphtheria, pertussis, and tetanus (DPT); at least three doses of polio vaccine; and one dose of measles.

Table 4.6: Summary of ITN use and basic vaccination coverage by state based on the model-based geostatistical predictions

| **State** | **MBG^1^** | **Modelled mean [95% Crl ] ITN use based on 2010 MIS** | **Estimated percentage population not using ITN based on MIS mean estimate** | **Estimated percentage [95% Crl ] of Children 12-23 months who received basic vaccination** | **Estimated percentage population not vaccinated** |
| --- | --- | --- | --- | --- | --- |
| Benue | 723,356 | 33.5 [22.0-45.9] | 66.5 | 35.4 [25.8-45.6] | 64.6 |
| Kwara | 444,502 | 35.9 [21.1-51.5] | 64.1 | 50.3 [38.8-61.8] | 49.7 |
| Nassarawa | 287,513 | 38.7 [25.2-52.8] | 61.3 | 29.6 [20.1-40.0] | 70.4 |
| Ondo | 522,906 | 37.7 [20.6-56.5] | 62.3 | 56.7 [45.0-68.0] | 43.3 |
| Delta | 679,112 | 41.9 [30.2-54.0] | 58.1 | 54.2 [42.4-65.9] | 45.8 |
| Jigawa | 970,793 | 71.3 [58.5-82.7] | 28.7 | 7.0 [3.4-11.7] | 93.0 |
| Bayelsa | 288,149 | 40.2 [25.0-55.8] | 59.8 | 37.2 [26.2-48.8] | 62.8 |
| Edo | 520,499 | 39.3 [26.5-52.7] | 60.7 | 52.2 [41.1-63.1] | 47.8 |
| FCT, Abuja | 207,728 | 47.4 [24.7-71.6] | 52.6 | 60.6 [48.9-72.0] | 39.4 |
| Rivers | 846,040 | 62.3 [50.4-73.5] | 37.7 | 50.1 [39.0-61.3] | 49.9 |
| Niger | 811,657 | 65.6 [53.8-76.7] | 34.4 | 28.1 [19.5-37.4] | 71.9 |
| Katsina | 1,370,240 | 39.3 [27.4-51.7] | 60.7 | 8.0 [4.1-12.9] | 92.0 |
| Abia | 409,348 | 40.9 [26.2-56.0] | 59.1 | 53.7 [41.4-65.7] | 46.3 |
| Akwa Ibom | 599,132 | 57.0 [44.7-69.0] | 43.0 | 52.9 [41.5-64.2] | 47.1 |
| Kaduna | 1,160,413 | 67.7 [56.9-77.9] | 32.3 | 36.7 [27.4-46.5] | 63.3 |
| Kebbi | 672,229 | 66.8 [52.5-80.1] | 33.2 | 17.6 [10.8-25.4] | 82.4 |
| Enugu | 474,033 | 38.7 [26.8-51.2] | 61.3 | 47.9 [36.4-59.5] | 52.1 |
| Zamfara | 797,087 | 48.0 [31.8-64.0] | 52.0 | 13.6 [8.0-20.6] | 86.4 |
| Taraba | 412,371 | 51.2 [35.3-66.6] | 48.8 | 31.6 [22.7-41.2] | 68.4 |
| Adamawa | 552,403 | 69.0 [57.5-79.6] | 31.0 | 31.0 [21.9-40.9] | 69.0 |
| Cross River | 489,666 | 51.9 [38.9-64.9] | 48.1 | 60.9 [49.8-71.4] | 39.1 |
| Osun | 486,478 | 36.7 [18.0-57.5] | 63.3 | 68.5 [57.1-79.1] | 31.5 |
| Anambra | 661,676 | 61.2 [49.1-72.7] | 38.8 | 65.4 [54.5-75.7] | 34.6 |
| Kano | 2,116,176 | 69.6 [59.9-78.5] | 30.4 | 16.7 [10.8-23.5] | 83.3 |
| Lagos | 1,638,486 | 36.9 [23.1-51.6] | 63.1 | 64.4 [54.4-73.9] | 35.6 |
| Gombe | 497,190 | 74.1 [62.8-84.2] | 25.9 | 26.9 [18.9-35.8] | 73.1 |
| Kogi | 508,698 | 42.4 [29.2-56.1] | 57.6 | 55.4 [43.7-66.9] | 44.6 |
| Borno | 890,870 | 63.1 [50.6-74.9] | 36.9 | 10.3 [5.6-16.0] | 89.7 |
| Bauchi | 966,465 | 62.7 [52.2-72.7] | 37.3 | 10.7 [5.8-16.7] | 89.3 |
| Imo | 613,111 | 38.0 [25.2-51.4] | 62.0 | 56.1 [44.2-67.6] | 43.9 |
| Ogun | 532,056 | 50.3 [33.0-68.0] | 49.7 | 38.2 [26.8-50.2] | 61.8 |
| Ebonyi | 321,430 | 46.2 [31.8-60.8] | 53.8 | 63.3 [52.4-73.6] | 36.7 |
| Sokoto | 787,264 | 67.7 [55.6-79.1] | 32.3 | 8.1 [4.0-13.3] | 91.9 |
| Ekiti | 340,160 | 39.2 [25.0-54.2] | 60.8 | 66.5 [55.8-76.5] | 33.5 |
| Plateau | 563,246 | 49.6 [36.4-62.8] | 50.4 | 44.8 [34.5-55.4] | 55.2 |
| Oyo | 958,289 | 33.8 [17.2-52.0] | 66.2 | 46.8 [35.9-57.9] | 53.2 |
| Yobe | 516,282 | 68.0 [55.0-79.8] | 32.0 | 11.3 [6.4-17.4] | 88.7 |
| **Total** | **25,637,054** | **50.9 [37.3-64.6]** | **47.2** | **39.7 [30.4-49.3]** | **60.3** |

1. Model-based geostatistics

Table 4.7 Summary of ITN use and basic vaccination coverage by state based on the census

| **State** | **Census** | **Mean [95% Crl ] ITN use based on 2010 MIS** | **Percentage population not using ITN based on MIS mean estimate** | **Percentage [95% Crl ] of Children 12-23 months who received basic vaccination** | **Percentage population not Vaccinated** |
| --- | --- | --- | --- | --- | --- |
| Benue | 848,955 | 33.5 [22.0-45.9] | 66.5 | 35.4 [25.8-45.6] | 64.6 |
| Kwara | 454,875 | 35.9 [21.1-51.5] | 64.1 | 50.3 [38.8-61.8] | 49.7 |
| Nassarawa | 366,688 | 38.7 [25.2-52.8] | 61.3 | 29.6 [20.1-40.0] | 70.4 |
| Ondo | 474,609 | 37.7 [20.6-56.5] | 62.3 | 56.7 [45.0-68.0] | 43.3 |
| Delta | 543,225 | 41.9 [30.2-54.0] | 58.1 | 54.2 [42.4-65.9] | 45.8 |
| Jigawa | 934,526 | 71.3 [58.5-82.7] | 28.7 | 7.0 [3.4-11.7] | 93.0 |
| Bayelsa | 209,525 | 40.2 [25.0-55.8] | 59.8 | 37.2 [26.2-48.8] | 62.8 |
| Edo | 450,683 | 39.3 [26.5-52.7] | 60.7 | 52.2 [41.1-63.1] | 47.8 |
| FCT, Abuja | 209,470 | 47.4 [24.7-71.6] | 52.6 | 60.6 [48.9-72.0] | 39.4 |
| Rivers | 650,050 | 62.3 [50.4-73.5] | 37.7 | 50.1 [39.0-61.3] | 49.9 |
| Niger | 853,465 | 65.6 [53.8-76.7] | 34.4 | 28.1 [19.5-37.4] | 71.9 |
| Katsina | 1,315,910 | 39.3 [27.4-51.7] | 60.7 | 8.0 [4.1-12.9] | 92.0 |
| Abia | 341,863 | 40.9 [26.2-56.0] | 59.1 | 53.7 [41.4-65.7] | 46.3 |
| Akwa Ibom | 502,131 | 57.0 [44.7-69.0] | 43.0 | 52.9 [41.5-64.2] | 47.1 |
| Kaduna | 1,246,660 | 67.7 [56.9-77.9] | 32.3 | 36.7 [27.4-46.5] | 63.3 |
| Kebbi | 696,628 | 66.8 [52.5-80.1] | 33.2 | 17.6 [10.8-25.4] | 82.4 |
| Enugu | 411,124 | 38.7 [26.8-51.2] | 61.3 | 47.9 [36.4-59.5] | 52.1 |
| Zamfara | 759,560 | 48.0 [31.8-64.0] | 52.0 | 13.6 [8.0-20.6] | 86.4 |
| Taraba | 429,724 | 51.2 [35.3-66.6] | 48.8 | 31.6 [22.7-41.2] | 68.4 |
| Adamawa | 569,816 | 69.0 [57.5-79.6] | 31.0 | 31.0 [21.9-40.9] | 69.0 |
| Cross River | 398,274 | 51.9 [38.9-64.9] | 48.1 | 60.9 [49.8-71.4] | 39.1 |
| Osun | 457,960 | 36.7 [18.0-57.5] | 63.3 | 68.5 [57.1-79.1] | 31.5 |
| Anambra | 520,335 | 61.2 [49.1-72.7] | 38.8 | 65.4 [54.5-75.7] | 34.6 |
| Kano | 1,987,150 | 69.6 [59.9-78.5] | 30.4 | 16.7 [10.8-23.5] | 83.3 |
| Lagos | 1,390,870 | 36.9 [23.1-51.6] | 63.1 | 64.4 [54.4-73.9] | 35.6 |
| Gombe | 486,338 | 74.1 [62.8-84.2] | 25.9 | 26.9 [18.9-35.8] | 73.1 |
| Kogi | 652,802 | 42.4 [29.2-56.1] | 57.6 | 55.4 [43.7-66.9] | 44.6 |
| Borno | 873,238 | 63.1 [50.6-74.9] | 36.9 | 10.3 [5.6-16.0] | 89.7 |
| Bauchi | 963,387 | 62.7 [52.2-72.7] | 37.3 | 10.7 [5.8-16.7] | 89.3 |
| Imo | 499,925 | 38.0 [25.2-51.4] | 62.0 | 56.1 [44.2-67.6] | 43.9 |
| Ogun | 502,121 | 50.3 [33.0-68.0] | 49.7 | 38.2 [26.8-50.2] | 61.8 |
| Ebonyi | 330,260 | 46.2 [31.8-60.8] | 53.8 | 63.3 [52.4-73.6] | 36.7 |
| Sokoto | 755,665 | 67.7 [55.6-79.1] | 32.3 | 8.1 [4.0-13.3] | 91.9 |
| Ekiti | 291,261 | 39.2 [25.0-54.2] | 60.8 | 66.5 [55.8-76.5] | 33.5 |
| Plateau | 579,497 | 49.6 [36.4-62.8] | 50.4 | 44.8 [34.5-55.4] | 55.2 |
| Oyo | 779,304 | 33.8 [17.2-52.0] | 66.2 | 46.8 [35.9-57.9] | 53.2 |
| Yobe | 475,848 | 68.0 [55.0-79.8] | 32.0 | 11.3 [6.4-17.4] | 88.7 |
| **Total** | **24,213,722** | **50.9 [37.3 - 64.6]** | 49.1 | **39.7 [30.4-49.3]** | 60.3 |

Table 4.8 Summary of ITN use and basic vaccination coverage by state based on the UN

| **State** | **UN** | **Mean [95% Crl ] ITN use based on 2010 MIS** | **Percentage population not using ITN based on MIS mean estimate** | **Percentage [95% Crl ] of Children 12-23 months who received basic vaccination** | **Percentage population not Vaccinated** |
| --- | --- | --- | --- | --- | --- |
| Benue | 809,219 | 33.5 [22.0-45.9] | 66.5 | 35.4 [25.8-45.6] | 64.6 |
| Kwara | 458,699 | 35.9 [21.1-51.5] | 64.1 | 50.3 [38.8-61.8] | 49.7 |
| Nassarawa | 336,242 | 38.7 [25.2-52.8] | 61.3 | 29.6 [20.1-40.0] | 70.4 |
| Ondo | 655,404 | 37.7 [20.6-56.5] | 62.3 | 56.7 [45.0-68.0] | 43.3 |
| Delta | 747,520 | 41.9 [30.2-54.0] | 58.1 | 54.2 [42.4-65.9] | 45.8 |
| Jigawa | 813,435 | 71.3 [58.5-82.7] | 28.7 | 7.0 [3.4-11.7] | 93.0 |
| Bayelsa | 294,605 | 40.2 [25.0-55.8] | 59.8 | 37.2 [26.2-48.8] | 62.8 |
| Edo | 624,612 | 39.3 [26.5-52.7] | 60.7 | 52.2 [41.1-63.1] | 47.8 |
| FCT, Abuja | 234,359 | 47.4 [24.7-71.6] | 52.6 | 60.6 [48.9-72.0] | 39.4 |
| Rivers | 958,439 | 62.3 [50.4-73.5] | 37.7 | 50.1 [39.0-61.3] | 49.9 |
| Niger | 753,034 | 65.6 [53.8-76.7] | 34.4 | 28.1 [19.5-37.4] | 71.9 |
| Katsina | 1,108,400 | 39.3 [27.4-51.7] | 60.7 | 8.0 [4.1-12.9] | 92.0 |
| Abia | 523,634 | 40.9 [26.2-56.0] | 59.1 | 53.7 [41.4-65.7] | 46.3 |
| Akwa Ibom | 718,837 | 57.0 [44.7-69.0] | 43.0 | 52.9 [41.5-64.2] | 47.1 |
| Kaduna | 1,139,910 | 67.7 [56.9-77.9] | 32.3 | 36.7 [27.4-46.5] | 63.3 |
| Kebbi | 605,223 | 66.8 [52.5-80.1] | 33.2 | 17.6 [10.8-25.4] | 82.4 |
| Enugu | 612,742 | 38.7 [26.8-51.2] | 61.3 | 47.9 [36.4-59.5] | 52.1 |
| Zamfara | 658,974 | 48.0 [31.8-64.0] | 52.0 | 13.6 [8.0-20.6] | 86.4 |
| Taraba | 403,781 | 51.2 [35.3-66.6] | 48.8 | 31.6 [22.7-41.2] | 68.4 |
| Adamawa | 566,638 | 69.0 [57.5-79.6] | 31.0 | 31.0 [21.9-40.9] | 69.0 |
| Cross River | 541,758 | 51.9 [38.9-64.9] | 48.1 | 60.9 [49.8-71.4] | 39.1 |
| Osun | 674,241 | 36.7 [18.0-57.5] | 63.3 | 68.5 [57.1-79.1] | 31.5 |
| Anambra | 805,489 | 61.2 [49.1-72.7] | 38.8 | 65.4 [54.5-75.7] | 34.6 |
| Kano | 1,801,330 | 69.6 [59.9-78.5] | 30.4 | 16.7 [10.8-23.5] | 83.3 |
| Lagos | 1,929,720 | 36.9 [23.1-51.6] | 63.1 | 64.4 [54.4-73.9] | 35.6 |
| Gombe | 438,003 | 74.1 [62.8-84.2] | 25.9 | 26.9 [18.9-35.8] | 73.1 |
| Kogi | 607,967 | 42.4 [29.2-56.1] | 57.6 | 55.4 [43.7-66.9] | 44.6 |
| Borno | 803,059 | 63.1 [50.6-74.9] | 36.9 | 10.3 [5.6-16.0] | 89.7 |
| Bauchi | 843,641 | 62.7 [52.2-72.7] | 37.3 | 10.7 [5.8-16.7] | 89.3 |
| Imo | 743,135 | 38.0 [25.2-51.4] | 62.0 | 56.1 [44.2-67.6] | 43.9 |
| Ogun | 625,931 | 50.3 [33.0-68.0] | 49.7 | 38.2 [26.8-50.2] | 61.8 |
| Ebonyi | 388,860 | 46.2 [31.8-60.8] | 53.8 | 63.3 [52.4-73.6] | 36.7 |
| Sokoto | 655,555 | 67.7 [55.6-79.1] | 32.3 | 8.1 [4.0-13.3] | 91.9 |
| Ekiti | 444,889 | 39.2 [25.0-54.2] | 60.8 | 66.5 [55.8-76.5] | 33.5 |
| Plateau | 592,722 | 49.6 [36.4-62.8] | 50.4 | 44.8 [34.5-55.4] | 55.2 |
| Oyo | 1,068,090 | 33.8 [17.2-52.0] | 66.2 | 46.8 [35.9-57.9] | 53.2 |
| Yobe | 441,401 | 68.0 [55.0-79.8] | 32.0 | 11.3 [6.4-17.4] | 88.7 |
| **Total** | **26,429,498** | **50.9 [37.3 - 64.6]** | 49.1 | **39.7 [30.4-49.3]** | 60.3 |

**References**
