# Supplementary material for: Fine resolution mapping of population age-structures for health and development applications
Source: J R Soc Interface. 2015 Apr 6;12(105):20150073. doi: 10.1098/rsif.2015.0073 (PMC4387535; doi:10.1098/rsif.2015.0073)
Supplement: Response to Reviewers.doc [file rsif20150073supp1.doc]

**Corresponding author**

Alegana Victor

Email: [vaa1g10@soton.ac.uk](mailto:valegana@kemri-wellcome.org)

**To**

**The Editor**

**Journal of the Royal Society Interface**

Re: **RSIF-2014-1070 (Fine resolution mapping of population age-structures for health and development applications)**

**Dear Editor**

We would like to thank you and the reviewers for the positive feedback and comments on the manuscript. We have made the recommended minor revisions, as outlined below:

**Referee: 1**

1. GENERAL REMARKS

**Comment:** *The paper reads much clearer than previously. I have a few minor comments. Otherwise I would recommend the submission for publication.*
*I much prefer the new scatter plots for Figures 4 & 5. I think you should say that they are ordered by census values.*

**Response:** We have included the suggestion on the caption.

**Comment:** *In black and white the choropleth maps are not clear because the red and blue look the same. Are they going to be printed in colour? If not then I suggest a `monotonic’ black and white scale.*
**Response:** They will be printed in colour online.

2. SPECIFIC REMARKS

1. p.4 l.49 Household is lower case: - **Corrected**
2. p.6 l.28 no comma after but - **Corrected**
3. p.8 Are we agreed on the use of `surface’ rather than something like `estimates’? – **Yes, replaced**
4. p.8 l.32 What were the small area methods used? **Response:** *These were used to estimate level of ITN and vaccination coverage at administration level 1 in Nigeria (details in the supplementary* information).

5. p.18 Table 3 Change mean and standard deviation to start with capitals - **Corrected**
6. p.21 Table 5 Remove `The highest gaps in vaccination…’ in the caption – **Corrected in figure 5**
7. p.21 Table 5 Include `for whole of Nigeria’ for consistency with other table – **Corrected in figure 5**
8. P21 Where is the equivalent proportion bednets plot? – **Added Figure 6**

**Referee: 2**

*The article has been much improved. I find it reads better and the graphs are an improvement. I would be happy to see it published, although the authors might want to correct a few minor errors (some jumped out; there must be others). E.g.:*

**Comment:**  *'To derived...' in the SI*

**Response**: Restructured the sentence ‘…A Bayesian linear mixed model was used to estimate ITN use at state level…’

**Comment:** *'Confidence intervals' in the captions for figs 4 & 5, which are presumably Bayesian prediction intervals.*

**Response**: Changed to Bayesian prediction interval

**Comment:** *And I found the new sentence 'Secondly, with the approach outlined here, the use of Gaussian white noise as the realisations of the unobserved levels of the proportions of the population that was under-five provided extra parametrisation.' hard to understand.*

**Response**: We have slightly modified this sentence to read: ‘.*.the Gaussian white noise specified in the SPDE approach adds extra parametrisation to the realisations of the unobserved levels of the proportions of the population...*’

**Comment:** *I am slightly disappointed the authors felt they could not make any sort of comparison with a non-spatial model though. I appreciate it is technically different, and this wasn't the aim of the piece, but the approach taken is couched as 'This is a better technique to use to predict these useful quantities'.*

We thank the review for comments. Yes, the objective of paper was not to compare the spatial approach to the non-spatial. We suggested the superiority of technique was based on the ability to incorporate prior effects (or prior knowledge) and model uncertainty. These are not usually employed in the non-Bayesian and non-spatial methods.

**Comment:** *Given that one could carry out a comparison of the out-of-sample predictive ability of their method and the non-spatial model (which would be far easier for other researchers and planners to implement themselves) I still think it would be interesting to know how much better the geospatial modelling performs.*

**Response:** We agree that a comparison of spatial and non-spatial approach can be useful in future applications. We also point out that model performance can be evaluated based on many other quantities rather than just out-of-sample predictions.

**Comment:** *To the extent that if the geospatial model predictions were worse, then I don't see why you would ever use it! Conversely, a much superior predictive ability would be a strong incentive to pick up this technique.*

**Response**: We believe, the choice of spatial methods should not be guided by quality of prediction but also presence of spatial structure (spatial autocorrelation) as illustrated in figure 1 (b). A variogram plot could also be used to check for spatial autocorrelation. It will not be useful to employ this approach in absence of spatial autocorrelation.

**Comment:** *I also stand by my point that not using numerator/denominator data loses information - unless the sampling is complete in a given location (perhaps it is here), you lose information about the precision with which the relevant proportion is known. But this is paragraph is irrelevant for review considerations.*

**Response**: We thank the review for observation. The DHS as pointed out in paper use a two-stage sampling scheme at cluster and household level. Perhaps incorporating uncertainty in the modelling framework is a useful step as demonstrated in the paper.
